# MMM: Integrative ensemble modeling and ensemble analysis

**DOI:** 10.1002/pro.3965

**Published:** 2020-10-17

**Authors:** Gunnar Jeschke

**Affiliations:** ^1^ ETH Zürich, Department of Chemistry and Applied Biosciences ETH Zürich Zürich Switzerland

**Keywords:** distance distributions, docking, ensemble modeling, integrative structural biology, protein complexes, RNA, site‐directed spin labeling

## Abstract

Proteins and their complexes can be heterogeneously disordered. In ensemble modeling of such systems with restraints from several experimental techniques the following problems arise: (a) integration of diverse restraints obtained on different samples under different conditions; (b) estimation of a realistic ensemble width; (c) sufficient sampling of conformational space; (d) representation of the ensemble by an interpretable number of conformers; (e) recognition of weak order with site resolution. Here, I introduce several tools that address these problems, focusing on utilization of distance distribution information for estimating ensemble width. The RigiFlex approach integrates such information with high‐resolution structures of ordered domains and small‐angle scattering data. The EnsembleFit module provides moderately sized ensembles by fitting conformer populations and discarding conformers with low population. EnsembleFit balances the loss in fit quality upon combining restraint subsets from different techniques. Pair correlation analysis for residues and local compaction analysis help in feature detection. The RigiFlex pipeline is tested on data simulated from the structure 70 kDa protein‐RNA complex RsmE/RsmZ. It recovers this structure with ensemble width and difference from ground truth both being on the order of 4.2 Å. EnsembleFit reduces the ensemble of the proliferating‐cell‐nuclear‐antigen‐associated factor p15^PAF^ from 4,939 to 75 conformers while maintaining good fit quality of restraints. Local compaction analysis for the PaaA2 antitoxin from *E. coli* O157 revealed correlations between compactness and enhanced residual dipolar couplings in the original NMR restraint set.

## 1 INTRODUCTION

Function of most proteins relies on a combination of rigid and flexible sections. For rigid sections, structure is defined at least at the resolution of chemical bond lengths, whereas flexible sections often adapt their conformation upon binding to other proteins, RNA, or small molecules. The flexible sections can undergo partial or complete disorder–order transitions.^1^ Such phenomena cannot be described in a narrow interpretation of Anfinsen's hypothesis,^2^ which assumes that amino acid sequence encodes a single conformer at atomic resolution. Much progress has been made recently in describing protein structure by conformational ensembles that rely on information from different experimental techniques.^3^ Yet, a systematic approach to generating representative ensembles of partially ordered proteins and their complexes is still elusive. The situation is especially unsatisfactory for assessing the width of the conformational ensemble. RNA‐binding proteins are a point in case, as they often feature extended disordered domains that are involved in promiscuous RNA binding^4^ as well as in formation of membrane‐less organelles by liquid–liquid phase separation.^5^

Here, I introduce a new ensemble modeling tool that is based on three established concepts:

- **Partitioning of the macromolecules** in rigid and flexible domains^6^
- **Utilizing ensemble width information** from nanometer‐range distance distributions,^7^, ^8^
- **Integrative structural biology**.^6^

The partitioning concept (i) drastically reduces the number of free parameters and thus improves sampling of relevant conformational space. The concept assumes that certain domains do conform to Anfinsen's hypothesis, which can be experimentally tested, for instance by NMR spectroscopy. In MMM, models with rigid domains joined by flexible linkers are built by the RigiFlex approach, which features another sampling advantage by factorizing conformational space into a subspace of rigid‐body arrangement and subspaces of individual flexible domains.

The distance distribution concept (ii) was introduced before for ensemble modeling of disordered protein domains^9^ and a brief account on a preliminary implementation of RigiFlex into MMM (Multiscale Modeling of Macromolecules) was given.^10^ Here, I introduce enumerated sampling of rigid‐body arrangements and building of flexible RNA sections.

The integrative structural biology concept (iii) is required since each nanometer distance distribution restraint (DDR) for a pair of spin labels requires preparation of one sample. This makes the DDRs sparse. Furthermore, because of flexibility of the label itself,^11^, ^12^, ^13^ DDRs are unsuited for determining the structure of rigid domains at high resolution. Finally, as DDRs are measured in the solid state, it is prudent to check whether they are consistent with data from techniques that can be applied in the physiologically more relevant liquid state. In particular, the new EnsembleFit module can simultaneously fit DDRs and small‐angle scattering (SAS) data.

This article is structured as follows. First, I describe the RigiFlex pipeline consisting of the Rigi, FlexRNA, Flex, and EnsembleFit modules. I explain enumerated sampling in Rigi, the FlexRNA algorithm, and scoring, sampling, and population fitting in EnsembleFit. Second, I introduce tools for analyzing heterogeneous order in conformation ensembles. Third, I describe tests of the RigiFlex pipeline and analysis modules on previously published ensembles. The Matlab®‐based, open‐source program MMM can be freely downloaded at www.epr.ethz.ch/software. The new tools are implemented in version 2020.2. Restraint files for the worked examples in the Supplementary Information are included in this distribution. The Supplementary Information describes the iterative clustering and sorting module SortGroup, illustrates output of the PairCorrelation module, and provides worked examples of using the new features of MMM as well as a brief description of restraint file conventions and keywords.

## 2 RIGIFLEX PIPELINE

**FIGURE 1:**
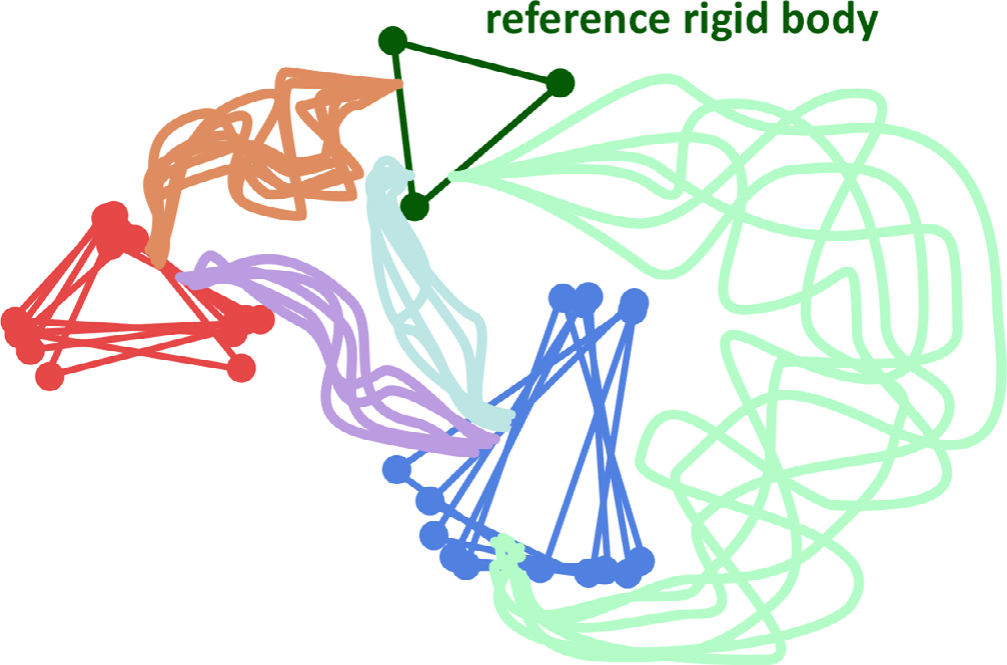
RigiFlex representation of a conformational ensemble. Each rigid body beyond the reference one (dark green) adds 6 free rotation and translation parameters, which are distributed. If three reference sites are selected per rigid body, the number of accessible pair distance distributions suffices for characterization of the distribution of rigid‐body arrangements (RBAs). Flexible peptide and RNA sections (pale shades) are added in a second step

**FIGURE 2:**
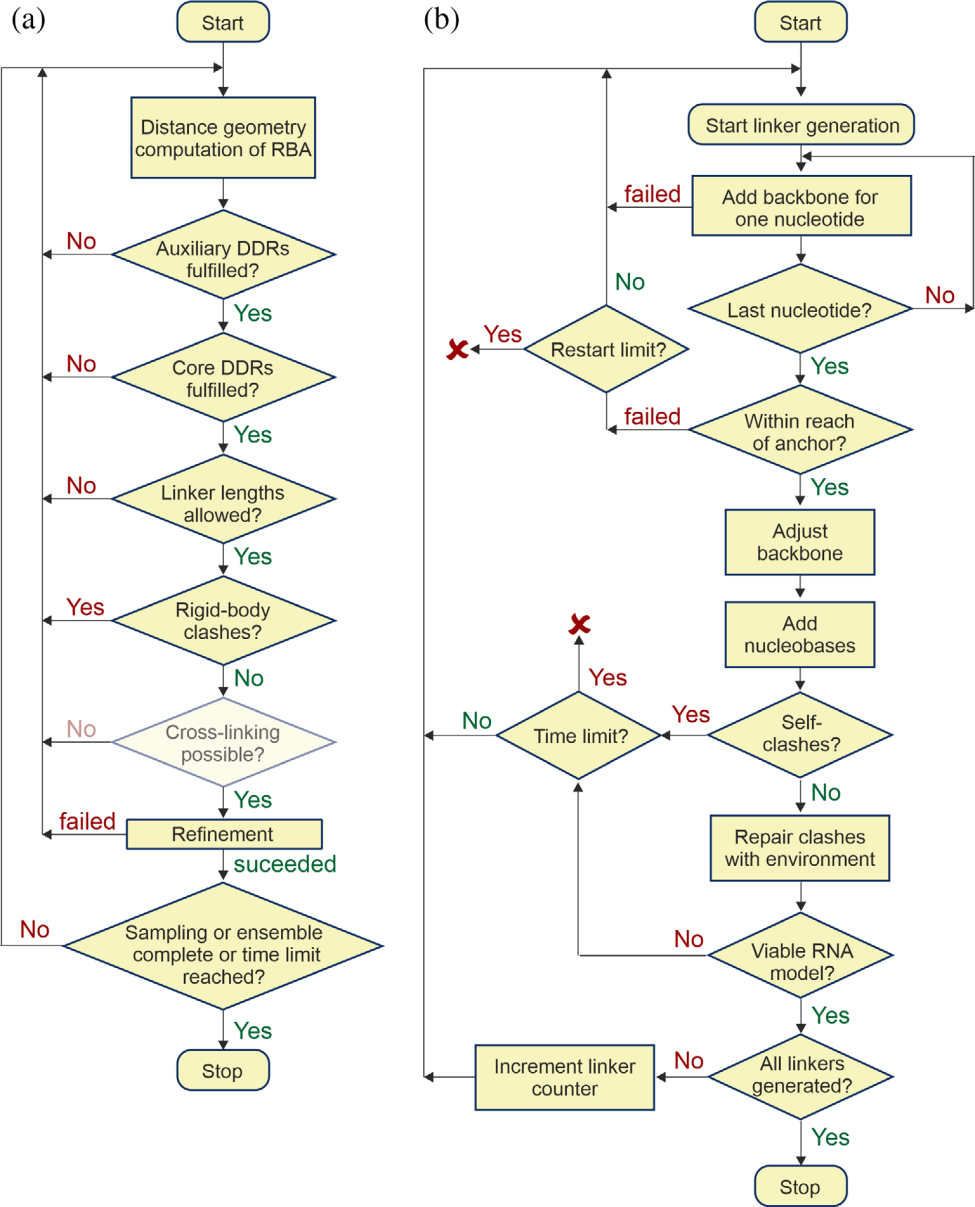
Flow charts for the Rigi module (a) and the FlexRNA module (b)

RigiFlex models proteins or their complexes by distributed arrangements of rigid bodies (triangles in Figure 1) joined by flexible linkers (pale lines in Figure 1). The first module Rigi performs enumerated sampling of distance matrices that conform to experimental distance distributions, computes rigid‐body arrangements (RBAs) by distance geometry,^14^ and samples and refines these RBAs by taking into account additional restraints (Figure 2a). The second module FlexRNA generates flexible single‐stranded RNA linkers based on a backbone pseudo‐torsion angle library.^15^ The third module Flex generates flexible peptide linkers by a previously established algorithm.^9^ The fourth module EnsembleFit scores the ensemble model against the full restraint set and improves this score by fitting populations of individual conformer models. In that process, ensemble size is reduced by discarding conformers with very low population. Finally, the remaining conformer models are sorted with respect to similarity by the SortGroup module described in Supplementary Information.

### 2.1 *The Rigi module*

We consider a protein or protein complex (entity) featuring a number *n* of bodies that are rigid on the resolution scale of their available atomic structures. Typically, such rigid bodies are parts of the entity that are resolved in an x‐ray or cryo‐EM structure or well defined in an NMR ensemble. RNA binding motifs can be part of a rigid body that consists mainly of protein domains.

The RBA is fully specified by 3(*n* − 1) translation and 3(*n* − 1) rotation parameters. Three reference sites per rigid body suffice for RBA determination via pair distances, as the number 9*n*(*n* − 1)/2 of accessible restraints exceeds 6(*n* − 1) for all *n* > 1.^10^ The optimal choice of the three reference sites in a rigid body are the vertices of the largest nearly equilateral triangle that can be realized, since this choice minimizes the potential for linear dependence of the reference DDRs. The problem is beyond a classical rigid‐body docking problem, as an ensemble of RBAs is sought that fits not only mean distances, but rather a set of distance distributions for reference point pairs.

**FIGURE 3:**
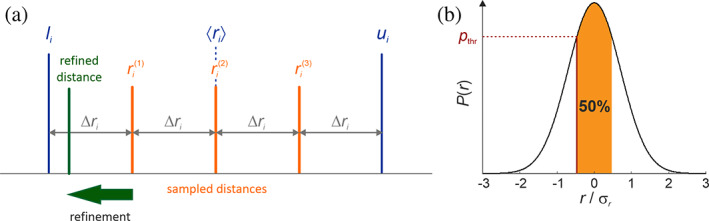
Processing of distance restraints in the Rigi module. (a) For each distance *r*_*i*_ between two reference points in different rigid bodies, *s*_*i*_ equidistant sampling points (*s*_*i*_ = 3 in the example) with restraint sampling resolution Δ*r*_*i*_ are distributed between a lower bound *l*_*i*_ and an upper bound *u*_*i*_. RBAs that fulfill all restraints at respective resolutions Δ*r*_*i*_ are refined and tested against a probability criterion. (b) The probability threshold *p*_thr_ rules acceptance of models with distances *r*_*i*,sim_. It is related to function values *g*_*i*_ = exp(−(*r*_*i*,sim_ − 〈*r*_*i*_〉)^2^/(2σ_*r*,*i*_^2^). The threshold *p*_thr_ is defined by probability percentage (here 50%) covered by values *g*_*i*_ ≥ *p*_thr_. Models are rejected if the geometric mean of all *g*_*i*_ is smaller than *p*_thr_. Note that *p*_thr_ is lower for a higher probability percentage

Rigi performs enumerated sampling of distance distributions instead of directly sampling the translation and rotation parameters. For each distance between two of the 3*n* reference sites, the samples are *s*_*i*_ points equidistant at restraint sampling resolution Δ*r*_*i*_ and situated in an interval between a lower bound *l*_*i*_ and an upper bound *u*_*i*_ (Figure 3a). For experimental restraints, specified by a mean distance 〈*r*_*i*_〉 and standard deviation σ_*r*,*i*_, I use *l*_*i*_ = 〈*r*_*i*_〉 − *σ*_*r*,*i*_, and *u*_*i*_ = 〈*r*_*i*_〉 + *σ*_*r*,*I*_, whereas for undetermined distances, I use a lower limit of 5 Å and a user‐defined upper limit that defaults to 180 Å. The number *s*_*i*_ of sampling points per restraint is selected by finding the minimal Δ*r* = max(Δ*r*_*i*_) under the constraint that the total number *T* (up to a few million) of distance restraint sets must fulfill the condition $T=\prod_{i=1}^{9n\left(n-1\right)/2}s_{i}\leq T_{\max}$. Since each distance restraint set defines a complete distance matrix for the 3*n* reference points, triangular bound smoothing^14^ can be applied in this optimization. By varying *T*_max_, the user can set a suitable Rigi sampling resolution Δ*r*.

RBAs that conform to the experimental distance distributions are generated by distance geometry^14^ for all *T* sets of sampled distances. Rigi then tests each RBA against further restraints in the order of increasing computational expense (Figure 2a). In particular, Rigi tests for auxiliary DDRs, where at least one labeling site is not a reference site, for a maximum length of peptide linkers of 3.8 Å per amino acid residue, for user‐specified maximum lengths of all RNA linkers (default: <7 Å per nucleotide), and for rigid‐body clashes. Simulated distances are converted to a fraction of the total distribution that still includes them (Figure 3b). An RBA is rejected if the geometric mean of all these fractions is above a user‐defined threshold.^9^ The default threshold of 0.5 corresponds to a mean coverage of 50% of the distributions. The user can further specify that a certain fraction 0 ≤ *f*_*x*_ ≤ 1 of crosslink restraints must be fulfilled in any accepted RBA.

If an RBA passes all tests at the sampling resolution Δ*r*, it is refined by optimization of the rotation and translation parameters. In order to prevent artificial narrowing of the ensemble, refinement stops as soon as all restraints are fulfilled, now without considering the sampling resolution as a contribution to uncertainty. Control of Rigi is explained in Supplementary Information.

### 2.2 *FlexRNA*

The FlexRNA module uses the same approach as Flex^9^ by replacing peptide backbone torsion angles by the pseudo‐torsion angles defined by Humphris‐Narayanan and Pyle.^15^ Their fragment library at 5° resolution^16^ and their algorithm for backbone generation are used. Figure 2b shows a flow chart of FlexRNA. To fix moderate misses in reaching the C3′‐terminal anchor nucleotide as well as moderate clashes with the environment, FlexRNA distributes the required stretch and rotation uniformly over the whole RNA backbone. This deformation is later relaxed by refining with Yasara.^17^ Linker generation can fail if no conformation is consistent both with the distance between the anchor nucleotides and with avoiding clashes with the rigid bodies. In order to avoid stalling of RigiFlex in such cases, the user can set runtime limits for FlexRNA and Flex. If not all flexible linkers can be generated for an RBA, the RBA is discarded.

Sampling resolution of the Flex and FlexRNA modules is not currently assessed separately. Instead, EnsembleFit (vide infra) predicts distance distributions for the whole ensemble. If these distance distributions are reasonably continuous and smooth and overlap well with the experimental distributions, sampling resolution is considered to be sufficient. A more sophisticated estimate of sampling resolution for stochastic sampling has been described.^18^

### 2.3 *The EnsembleFit module*

**FIGURE 4:**
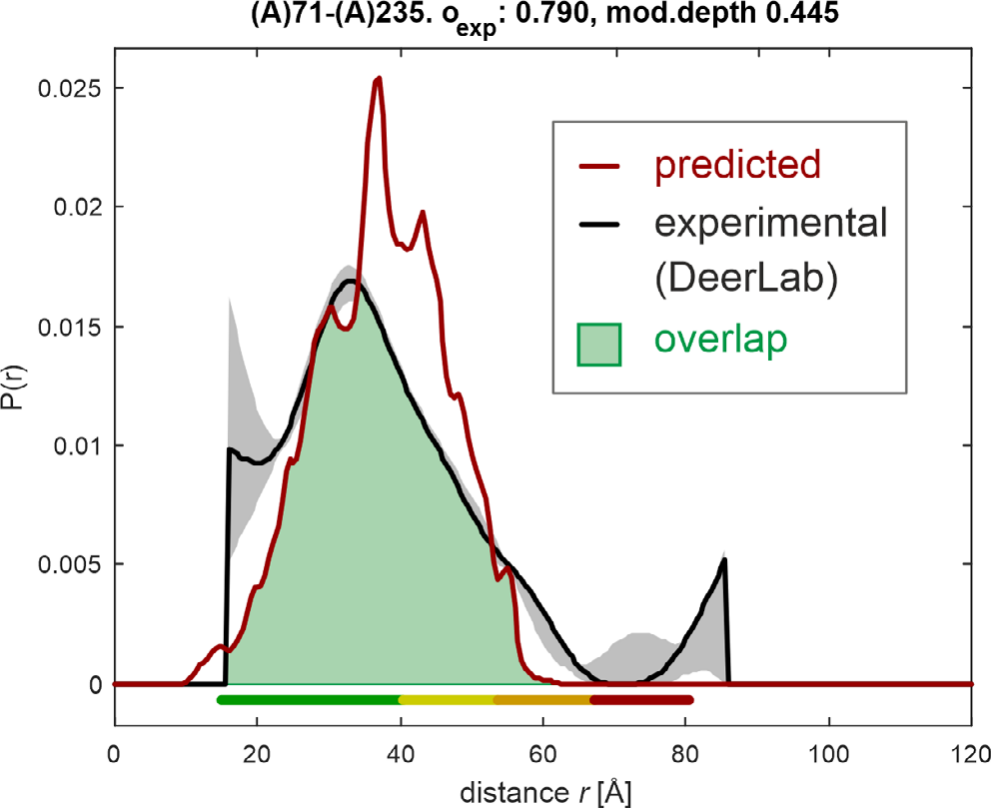
Definition of overlap for distance distribution restraints. The experimental distance distribution ***P***_DDR_ (black) and the distribution predicted for the ensemble ***P***_pred_ (red) are normalized to unit area. The fraction of overlapping area (green) is a measure for agreement of the ensemble with the restraint. Primary data were taken from the thesis of Christoph Gmeiner^19^ on the PTBP1/EMCV‐IRES DtoF complex and reprocessed with DeerLab.^20^ The colored bar below the distribution encodes reliability of the distribution. Shape is reliable in the range marked green, width is still reliable in the yellow range, mean distance still reliable in the orange range, and the presence of some contributions can still be ascertained in the red range. Modulation depth (mod. depth) is one characteristic of sample quality

Description by a conformational ensemble aims at functional realism, as we want to understand how the entity performs tasks within a cell. Unfortunately, we cannot generally verify functional realism. Instead, we have to be content with a description that is in line with all experimental information—as far as that is possible—and parsimonious. With parsimony, we run the risk of underestimating the true width of the ensemble.^9^ The RigiFlex approach contains this risk by fitting not only the mean distances but also distribution widths and shapes. Populations *p*_*j*_ are assigned to individual conformer models and are varied in order to find the best‐fit ensemble. To that end, the EnsembleFit module maximizes overlap *o*_*d*_ =  ∑ min{***P***_*pred*_, ***P***_*DDR*_} of the distance distribution ***P***_*pred*_ predicted for the ensemble model and the experimental distance distribution ***P***_*DDR*_ (Figure 4), taking into account the whole ensemble of conformer models. By maximizing the geometric mean of overlaps *o*_*i*_ of all DDRs indexed by *i*, fitting strongly penalizes small overlap of individual DDRs.

Such fitting of populations is straightforward if experimental errors are purely statistical and if the same score, preferably *χ* ^2^ values, can be applied for all restraints. In practice, integrative structural biology relies on data from different techniques, performed on different sample preparations under different conditions. Systematic errors are not negligible and models for predicting data from structure are imperfect. This complicates weighting of deviations between the different techniques and introduces poorly quantified sources of uncertainty into Bayesian approaches. In order to address this problem, EnsembleFit first separately fits subsets of restraints that share the same score metric (homogeneous restraints). Second, it combines the subsets by balancing loss in fit quality between them.

Given *N*_*v*_ valid conformers, in a first step vectors ***p*** ^(*k*)^ of populations *p*_*j*_^(*k*)^ (*j* = 1… *N*_*v*_) are fitted by minimizing some measure *m*_1_^(*k*)^ for the fit deviation of only the *k* ^th^ subset of restraints (*k* = 1… *R*, where *R* is the number of restraint subsets with different metrics). For example, if both DDRs and small‐angle scattering (SAS) restraints are available, we have *R* = 2 and define ${m_{1}^{\left(1\right)}=m}_{1}^{\left(\mathrm{DDR}\right)}=1-{\left(\prod_{i=1}^{D}o_{i}\right)}^{1/D},$ where the *o*_*i*_ are the overlaps for *D* DDRs, and ${m_{1}^{\left(2\right)}=m}_{1}^{\left(\mathrm{SAS}\right)}=\sum_{i=1}^{S}\chi_{i}^{2}$, where the $\chi_{i}^{2}$ are the *χ* ^2^ values for *S* SAS curve fits. Population vectors ***p*** ^(1)^ and ***p*** ^(2)^ generally differ.

In a second step, the final population vector ***p*** is fitted by minimizing the loss of merit, $L=\frac{1}{R}\sum_{k=1}^{R}\frac{m_{2}^{\left(k\right)}}{\;m_{1}^{\left(k\right)}}-1$, where the $m_{2}^{\left(k\right)}\;$ follow the same definition as the $m_{1}^{\left(k\right)}$, but relate to ***p*** rather than to the ***p*** ^(*k*)^. Only if all *R* restraint subsets were perfectly consistent, the vectors ***p*** ^(*k*)^ would all be identical and we would have *L* = 0. If they are somewhat inconsistent, normalization by the individual $m_{1}^{\left(k\right)}$ ensures that they are weighted according to their quality. This weighting still depends on the exact definition of the $m_{1}^{\left(k\right)}$, but it does take into account systematic measurement errors and prediction errors. Furthermore, the loss of merit *L* is a measure for inconsistency of the restraint subsets.

The global minima of the $m_{1}^{\left(k\right)}$ and of *L* can be found with reasonable computational expense for up to about *N*_*B*_ = 100 conformer models. The total number *N*_*c*_ of conformer models of the RigiFlex pipeline at the input of EnsembleFit can be much larger. This problem is solved by adhering to the principle of parsimony and by an iterative approach. After minimizing *L* for a block of *N*_B_ conformers, all conformers with *p*_*i*_ < 0.01·max(*p*_*i*_) are discarded. Often, many of the *p*_*i*_ approach zero during fitting. Removed conformers are then replaced by previously untested conformers to fill to the original block size *N*_*B*_. This process is repeated until no untested conformers are left. Dependence of the result on block size is weak if the number of conformers with *p*_*i*_ > 0.01 max(*p*_*i*_) is significantly smaller than block size. Larger block sizes up to about 250 can be used, albeit at the expense of longer computation times for the same total number *N*_*c*_ of conformers. The final ensemble is described by *N* conformers and their populations 0 ≤ *p*_*c*_ ≤ 1 (*c* = 1… *N*, ∑_*c*_ *p*_*c*_ = 1).

The current implementation of EnsembleFit processes only the two subsets of restraints mentioned above, DDRs and SAS curves, with the $\chi_{i}^{2}$ values being computed by CRYSOL (small‐angle X‐ray scattering) or CRYSON (small‐angle neutron scattering) of the ATSAS package.^21^ Implementation of restraint subsets for other techniques requires a module that predicts experimental data for a single conformer and a definition of the metric $m_{1}^{\left(k\right)}$.

In the original output ensemble of EnsembleFit, the *N* conformers appear in no particular order. The additional tool SortGroup, described in Supplementary Information, sorts and groups conformers by similarity.

EnsembleFit does not rely on raw ensembles generated by RigiFlex. It can also process unrestrained ensembles generated by flexible‐Meccano^22^ or TraDES^23^ or restrained ensembles generated by CYANA.^24^ In that sense, EnsembleFit is an alternative to ASTEROIDS^25^ or ENSEMBLE,^26^ which can take advantage of distance distribution information. Unlike these tools, EnsembleFit cannot yet utilize NMR restraints.

## 3 ENSEMBLE ANALYSIS

Two new tools in MMM serve for characterizing heterogeneous disorder. PairCorrrelation is suitable for revealing a low extent of disorder while LocalCompaction can reveal a small extent of order. With the Cα root mean square deviation *D*_*ij*_ of conformers *i* and *j* i upon their optimal superposition, we define an ensemble width

(1) $$\Gamma =\sqrt{\frac{\sum_{i=1}^{N-1}\sum_{j=i+1}^{N}p_{i}p_{j}D_{\mathrm{ij}}^{2}}{\sum_{i=1}^{N-1}\sum_{j=i+1}^{N}p_{i}p_{j}}}$$

as well as a distance between two ensembles *E*_1_ and *E*_2_

(2) $$\Gamma_{E_{1},E_{2}}=\sqrt{\frac{\sum_{S_{i}\in E_{1}}\sum_{S_{j}\in E_{2}}p_{i}p_{j}D_{\mathrm{ij}}^{2}}{\sum_{S_{i}\in E_{1}}\sum_{S_{j}\in E_{2}}p_{i}p_{j}}}$$

where the two sums run over all conformers in *E*_1_ and *E*_2_, respectively. The distance $\Gamma_{E_{1},E_{2}}$ defined in this way cannot be expected to be lower than the geometric mean of the two ensemble widths.

### 3.1 *The PairCorrelation module*

We consider Cα distances $r_{\mathrm{mn}}^{\left(j\right)}$ for a pair of residues with indices *m* and *n* in structures with index *j*. These distances have a mean value 〈*r*_*mn*_〉 and a standard deviation *σ*(*r*_*mn*_). The standard deviation *σ*(*r*_*mn*_) and relative standard deviation *σ*(*r*_*mn*_)/〈*r*_*mn*_〉 are measures for the distribution of the Cα‐Cα distance between residues *m* and *n*. Values of zero denote perfect order, as expected, for instance, within the same rigid body. A colored matrix representation of the *σ*(*r*_*mn*_) reveals residue pairs whose motion may be correlated in the conformational dynamics that underlies the ensemble. Two examples are given in the Supplementary Information.

### 3.2 *The LocalCompaction module*

Compactness of a section of a random coil between residues *m* and *n* (*m* < *n*) is quantified by its radius of gyration

(3) $$R_{g}^{\left(m,n\right)}=\sqrt{\frac{1}{n-m+1}\sum_{k=m}^{n}{\left(r_{k}-r_{c}\right)}^{2}},$$

where

(4) $$r_{c}=\frac{1}{n-m+1}\sum_{k=m}^{n}r_{k}$$

is the center coordinate of the section. For comparing the radius of gyration to SAS data, *k* must run over all atoms. For ensemble analysis, it suffices to consider the Cα atoms. Flory theory predicts for a random coil

(5) $$R_{g}^{\left(m,n\right)}=R_{0}{\left(n-m\right)}^{\nu },$$

where *R*_0_ is a segment length and exponent ν quantifies compactness. The range for *ν* extends from 0.33 for a collapsed coil in a poor solvent to 0.6 for an extended coil in a good solvent. The latter value has been found to good approximation in experimental^27^ and a computational^28^ studies for chemically unfolded proteins.

In an ensemble on *N* conformers with *n*_res_ residues each, LocalCompaction fits Equation (5) globally to *N*·*n*_res_·(*n*_res_ − 1)/2 segments by defining an ensemble average that scales linearly with *n*_res_ for an ideal chain

(6) $$\sqrt{〈{\left(R_{g}^{\left(m,n\right)}\right)}^{2}〉}=\sqrt{\sum_{i=1}^{N}p_{i}{\left(R_{g,i}^{\left(m,n\right)}\right)}^{2}}.$$

The symmetric matrix **G** with elements

(7) $$G_{\mathrm{nm}}=\frac{\sqrt{〈{\left(R_{g}^{\left(m,n\right)}\right)}^{2}〉}-R_{0}{\left(n-m\right)}^{\nu }}{R_{0}{\left(n-m\right)}^{\nu }}.$$

quantifies deviation of the radius of gyration of each chain segment from a mean random‐coil description of the whole chain.

This concept can be extended to a more intuitive proximity matrix **P**. For a random coil in an ideal (theta) solvent (*ν* = 0.5), we have $〈R^{2}〉=6R_{g}^{2}$. For good (*ν* > 0.5) or poor (*ν* < 0.5) solvents, I empirically assume that $\sqrt{〈R^{2}〉}$ has the same scaling behaviour as $\sqrt{〈{R_{g}}^{2}〉}$. Local Compaction performs a global fit of the root mean square end‐to‐end distance of segments $\sqrt{〈R_{\mathrm{mn}}^{2}〉}$ from residue *m* to *n* to a Flory equation, $\sqrt{〈R_{\mathrm{mn}}^{2}〉}=R_{0,\mathrm{ee}}{\left(n-m\right)}^{\nu_{\mathrm{ee}}},$ where *R*_0,*ee*_ is an effective segment length and *ν*_*ee*_ a scaling exponent. Matrix elements *P*_*mn*_ of the proximity matrix **P** can then be defined in analogy to Equation (7). This proximity matrix **P** is more sensitive to local compaction or expansion than the compactness matrix **G**.

## 4 TESTS

### 4.1 *RigiFlex pipeline*

**FIGURE 5:**
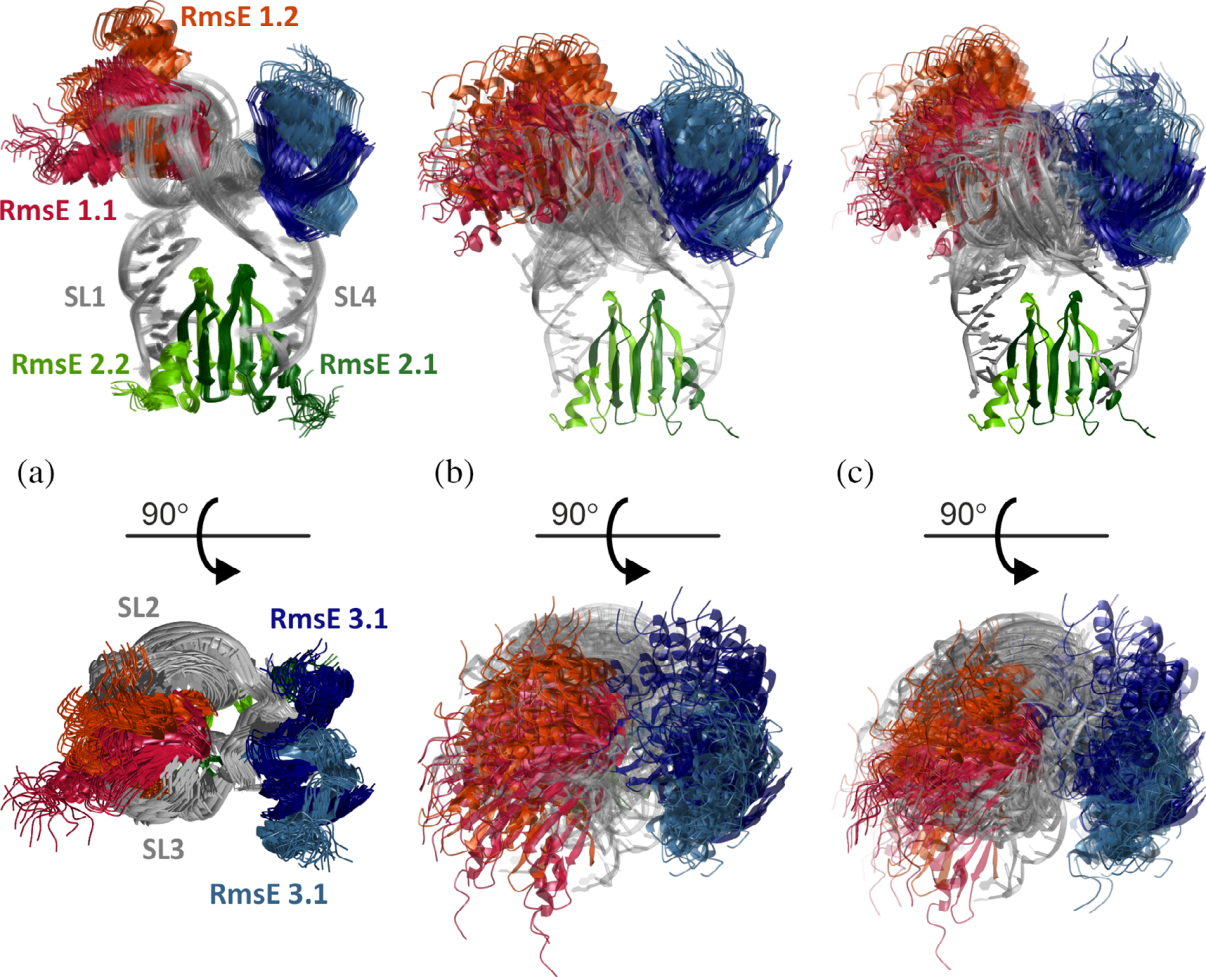
Cartoon plots of ensemble models for conformer R of RsmE/RsmZ. The models are superimposed on the RsmE homodimer in rigid body 2 (dark green/light green). The other RsmE homodimers are colored crimson/orange red (rigid body 1) and dark blue/steel blue (rigid body 3), whereas RNA is colored grey. (a) Ground‐truth ensemble stemming from a CYANA computation with experimental restraints (PDB 2MF1, 20 models, ensemble width *Γ*_1_ = 1.85 Å).^19^ (b) Small raw ensemble recomputed with RigiFlex from simulated DDRs (21 models, ensemble width *Γ*_2_ = 4.26 Å). (c) Representative ensemble generated by EnsembleFit from a RigiFlex raw ensemble with 224 models using the same DDRs (30 models, ensemble width *Γ*_3_ = 4.30 Å). Populations are encoded by transparency, with the most populated model shown fully opaque

As test case for the RigiFlex pipeline, I use the 70 kDa complex of three dimers of the translation‐repression protein RsmE with the first 72 nucleotides of the small RNA RsmZ that can sequester RsmE and thus de‐repress translation initiation. Structures of two conformers of this complex had been originally modeled by CYANA^24^ based on NMR restraints for the RsmE dimer, the first four stemloops (SL) of RsmZ, and a short GGA binding motif in the linker between SL2 and SL3 as well as on 21 DDRs between RNA labeling sites.^29^ The ensemble of 20 models of conformer R of the RsmE/RsmZ complex (PDB 2MF1) is considered here as the ground truth. Rigid bodies were extracted from model 1 of the ensemble. Since the original restraint set does not conform to the RigiFlex approach, I assigned valines 8 and 40 in loop regions of the first RsmE protomer and valine 40 in the second protomer of a dimer as reference sites and valine 8 in the second protomer as an auxiliary site. Using rotamer library modeling in MMM, I computed 18 reference DDRs involving two reference sites and 6 auxiliary DDRs involving one reference site and one auxiliary site. I encoded them as Gaussian restraints. The restraint file is distributed with MMM 2020.2. For a first test, I specified a maximum of *T*_max_ = 20′000 trials for exhaustive search of RBA space. As the distance distributions computed from the ground‐truth ensemble are narrow, this leads to a sampling resolution Δ*r* as good as 3.4 Å with *T* = 17′496 trials. This run provided 25 RBAs, of which 21 could be linked by FlexRNA. Figure 5a,b demonstrates that the width of this ensemble (Γ = 4.26 Å) is larger than the one of the ground truth ensemble (Γ = 1.85 Å). The distance from the ground truth ensemble, Γ_a,b_ = 4.20 Å, exceeds the geometric mean of the two ensemble widths (2.81 Å), but appears acceptable given the uncertainty of about 2–3 Å in rotamer simulations of label‐to‐label distances.^12^, ^13^

As a second test, I generated a moderately sized raw ensemble of the RsmE/RsmZ complex with *T* = 311 040 trials in Rigi (sampling resolution Δ*r* = 3.1 Å). Of the 301 RBAs found in this run, 224 could the linked by FlexRNA. Using EnsembleFit, I reduced this raw ensemble to a representative ensemble of *N* = 30 conformers. This ensemble (Figure 5c) has about the same width (Γ = 4.30 Å) as the small raw ensemble generated by Rigi and FlexRNA without ensemble fitting (Figure 5b). It slightly better matches the ground truth (Γ_a,c_ = 4.08 Å). The limited resolution resulting from the uncertainty of the spin label positions cautions against using this approach for structure determination of highly ordered systems.

### 4.2 *EnsembleFit*

As a test for using the EnsembleFit module on ensembles generated by other approaches, I reduced the ensemble of the highly disordered 111‐residue‐long proliferating‐cell‐nuclear‐antigen (PCNA)‐binding protein p15^PAF^, which is based on NMR and SAXS information.^30^ For the 4,936 conformers of ensemble PED6AAA from the protein ensemble database,^31^ I simulated 21 DDRs for all site pairs in the set V2, V17, S35, C54, L71, S88, and L101 and estimated uncertainty of the DDRs by separating the ensemble into two subensembles with 2,470 and 2,469 conformers. For the complete ensemble, I found imperfect agreement between the SAXS curve predicted by CRYSOL (version 3.0.1 of ATSAS)^21^ and the experimental curve (*χ* ^2^ = 3.053). As the SAXS curve could be fitted well with small subsets of conformers, I first fitted only this curve by optimizing populations for 49 individual blocks of 100 conformers and a final block of 39 conformers. The 50 subsensembles contained 135 conformers. Assuming uniform populations, they fit the experimental SAXS curve with *χ* ^2^ = 1.294 and the DDRs with a mean overlap of 0.897.

**FIGURE 6:**
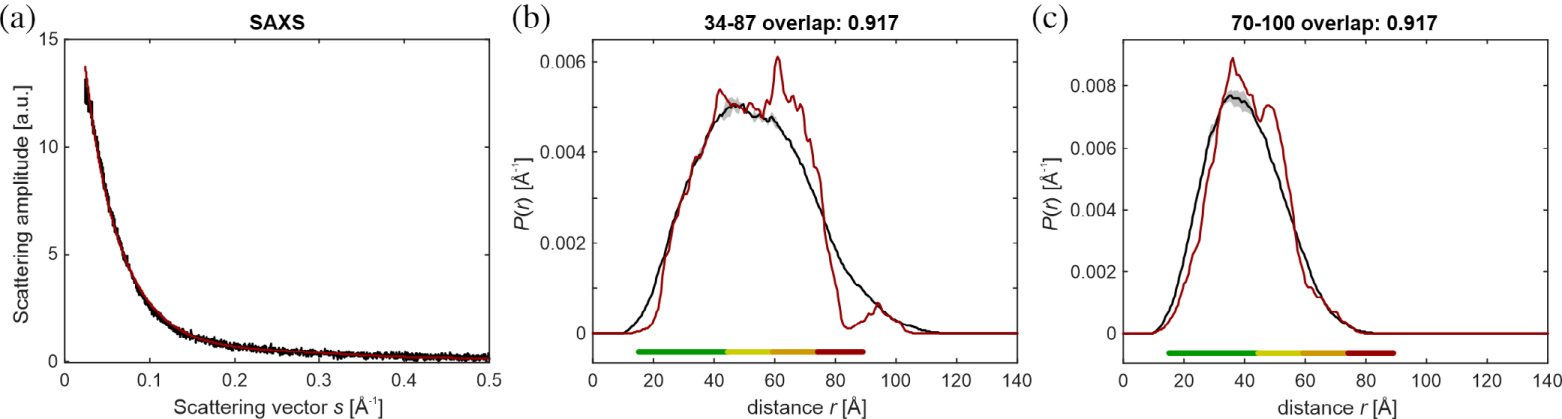
Restraint fits for the representative ensemble of 75 conformers reduced from the original NMR/SAXS ensemble of p15PAF (4,939 conformers)^30^ by a two‐step approach using the original SAXS curve and simulated DDRs (see text). Shown are the fit of the SAXS curve (a) by CRYSOL^21^ with *χ* ^2^ = 1.241 and the distance distribution fits for the two DDRs with the worst overlaps (b,c) between ground‐truth distance distribution (black with grey confidence bands) and the distribution for the ensemble (crimson). The colored reliability bars (see Figure 5 for explanation) refer to putative experimental DEER data of 8 μs length

I then treated these 135 conformers as a single block and fitted to the DDRs and the SAXS curve simultaneously. The resulting ensemble with 75 conformers had a SAXS *χ* ^2^ of 1.241, a DEER overlap of 0.940, and a loss of merit *L* = 0.088, indicating good consistency between the restraint subsets for the strongly reduced ensemble. Figure 6 shows that this ensemble fits the SAXS curve reasonably well and that even for the two DDRs with the worst overlap of 0.917, mean, width, and shape of the distance distributions match quite well.

### 4.3 *LocalCompaction*

**FIGURE 7:**
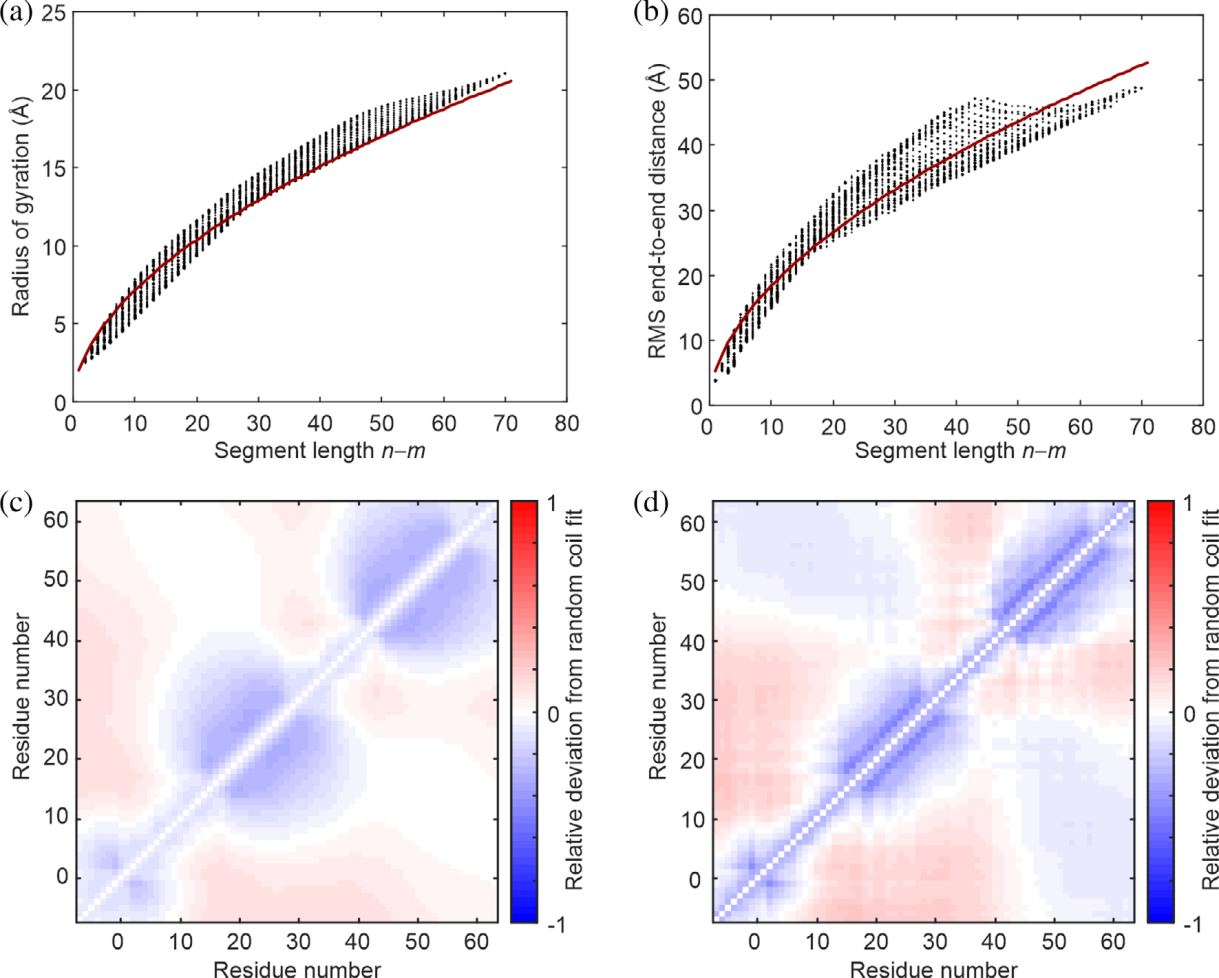
Compactness (a,b) and proximity (c,d) analysis of the NMR/SAXS ensemble of PaaA2.^32^ (a) Distribution of segment radii of gyration in the ensemble (black dots) as a function of segment length and fit by a random‐coil model (crimson line) with *R*_0_ = 2.07 Å and *ν* = 0.538. (b) Compactness matrix **G**. Blue shades mark segments that are more compact than expected from the random‐coil fit, red shades those that are more extended. (c) Distribution of segment root mean square end‐to‐end distances as a function of segment length (black dots) and fit by a random‐coil model (crimson line) with *R*_0,ee_ = 5.31 Å and *ν*_ee_ = 0.538. (c) Proximity matrix **P**. Blue shades mark segments that are on average shorter than expected from the random‐coil fit, red shades those that are on average longer

The LocalCompaction module was tested on the NMR/SAXS ensemble of PaaA2 antitoxin from *E. coli* O15734 (PED5AAA),^32^ which is highly flexible, but contains two preformed helices. The random‐coil fit provides *ν* = 0.538, corresponding to a somewhat more compact ensemble than is observed for chemically unfolded proteins (Figure 7a). Furthermore, the two preformed helices are clearly discernible in **G** as compact segments (blue shades in Figure 7b).

As seen by comparing Figure 7a,b, root mean square end‐to‐end distances are more broadly distributed at given segment length than are the radii of gyration. The scaling exponent *ν*_ee_ = 0.538 for $\sqrt{〈R_{\mathrm{mn}}^{2}〉}$ is identical to the one for $\sqrt{〈{\left(R_{g}^{\left(m,n\right)}\right)}^{2}〉}$ by coincidence. In the proximity matrix **P** (Figure 7d), the two preformed helices are better defined than in matrix **G** and the degree of compaction or extension between segments of the protein is better visible.

## 5 CONCLUSION

Proteins and their complexes are often neither completely structured nor completely unstructured. The exhibit semistructure with an extent of order that varies between domains or even along peptide or nucleic acid chains within the same domain. Such semistructured entities must be represented by ensembles. The ensembles are based on restraints from different experimental techniques that are performed with different sample preparation and under different conditions. The restraints may thus not be fully consistent. Here, I introduced several tools for generating and analyzing ensembles that represent all subsets of experimental data weighted by their quality.

In particular, the RigiFlex approach models proteins and their complexes in terms of distributed arrangements of rigid bodies connected by flexible linkers. The EnsembleFit module integrates restraint subsets from different techniques by balancing loss in fit quality when going from fits of subsets to fits of all restraints. EnsembleFit generates moderately sized ensembles by fitting populations. Both RigiFlex and EnsembleFit are intended for combining distance distribution restraints with other types of restraints in integrative structure modeling. Ensemble models obtained by the RigiFlex pipeline or by other approaches can be analyzed for weak disorder or weak order effects by the PairCorrelation and LocalCompaction modules, respectively.

I hope that these tools provide further inroads into the advancing field of ensemble modeling. RigiFlex and EnsembleFit are currently being extended to further types of experimental restraints.

## AUTHOR CONTRIBUTIONS

**Gunnar Jeschke:** Conceptualization; data curation; formal analysis; funding acquisition; investigation; methodology; project administration; resources; software; validation; visualization; writing‐original draft; writing‐review and editing.

## Supporting information


**Appendix** **S1**: Supporting Information
