# Supplementary material for: MMM: Integrative ensemble modeling and ensemble analysis
Source: Protein Sci. 2020 Oct 17;30(1):125–35. doi: 10.1002/pro.3965 (PMC7737775; doi:10.1002/pro.3965)
Supplement: Supplementary file 1 — Appendix S1: Supporting Information [file PRO-30-125-s001.pdf]

Supplementary Information MMM: Integrative Ensemble Modelling and Ensemble Analysis

Gunnar Jeschke[&]

***Abstract*—This Supplementary Information describes the SortGroup module for sorting conformers by similarity, illustrates output of the PairCorrelation module, and provides some worked examples related to figures in the main text. In addition, it describes the format of MMM restraint files.**

I. The SortGroup module

For each pair (i, j) of conformer structures S_i and S_j, $(i, j = 1 \ldots N)$, the Cα root mean square deviation D_{ij} upon optimal superposition[1] is computed. For nucleic acids, C4' atom coordinates are used instead of Cα coordinates. The D_{ij} are then arranged in an $N \times N$ dissimilarity matrix $\mathbf{D}$. By hierarchical clustering in Matlab, natural divisions in the ensemble are identified. To that end, SortGroup starts with an inconsistency coefficient[2] of 1.5 and lowers it by decrements of 0.01 until more than one cluster is found. Total population P_k of a cluster C_k is the sum of the populations p_j for all $S_j \in C_k$. The cluster with the largest P_k is assigned as the first cluster (C'_1 after reordering).

The distance between two clusters k and l is defined as

$$\sigma_{kl} = \sqrt{\frac{\sum_{S_i \in C_k} \sum_{S_j \in C_l} p_i p_j D_{ij}^2}{\sum_{S_i \in C_k} \sum_{S_j \in C_l} p_i p_j}} \,. \quad (1)$$

The clusters are now sorted in ascending order of the σ_{1l}. This process is repeated recursively within each cluster that contains more than two conformers. The success of such cluster sorting can be judged by a color representation of the dissimilarity matrix $\mathbf{D}'$. For the RigiFlex/EnsembleFit ensemble of RsmE/RsmZ conformer R with 30 models, grouping into small subensembles of similar models is very apparent (Figure 1a). For the strongly reduced ensemble of p15PAF (Figure 1b), closer inspection still reveals subensembles of between two and five similar conformers, while there exist a few conformers with Cα RMSD of more than 10 Å with respect to any other conformer.

II. The PairCorrelation module

Output of the PairCorrelation module is visualized in Figure 2. In the pair correlation matrix of RsmE/RsmZ based on standard deviation $\sigma(r_{mn})$ (panel (a)), rigid bodies are recognized as blue squares and rectangles. The sites of largest structural variation (yellow) mostly involve termini of RsmE in rigid body 2. The flexible RNA linker between stemloops 2 and 3, which connects rigid bodies 1 and 3, also shows enhanced variation with respect to rigid body 2.

[&]ETH Zürich, Department of Chemistry and Applied Biosciences, Vladimir-Prelog-Weg 2, CH-8093 Zürich, Switzerland. gjeschke@ethz.ch

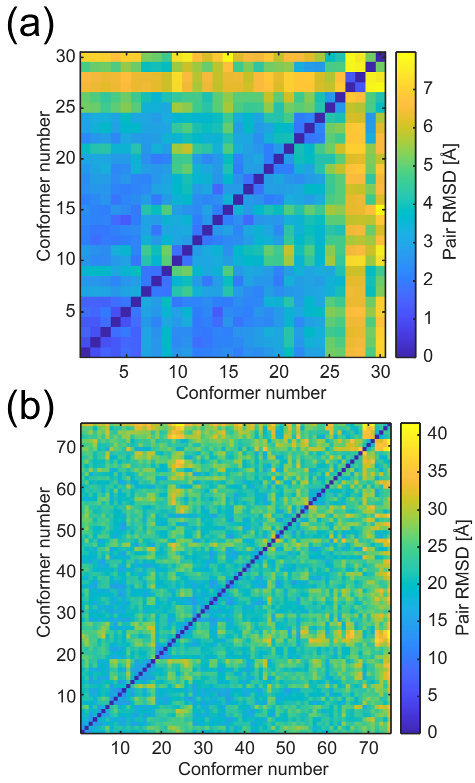

Fig. 1. Ensemble sorting for RsmE/RsmZ [3] (a) and for the representative ensemble of p15PAF [4] (b).

For less ordered domains, relative standard deviation $\sigma(r_{mn})/r_{mn}$ is better suited (Figure 2(b)). For the NMR/SAXS ensemble of PaaA2 antitoxin from E. coli O15734 (PED5AAA), correlation between local order and the NMR N-HN residual dipolar couplings (RDCs) is clearly visible for the two preformed helices Glu18 to Lys28 and His42 to Arg57. Such correlation is also apparent between preformed helix 42-57 and the N-terminal FLAG tag of the studied construct.

III. Ensemble modelling of RsmE/RsmZ

A. Generation of the small Rsme/RsmZ ensemble by RigiFlex

In the Matlab command window, change to the MMM subdirectory `demo\RsmE_RsmZ` and

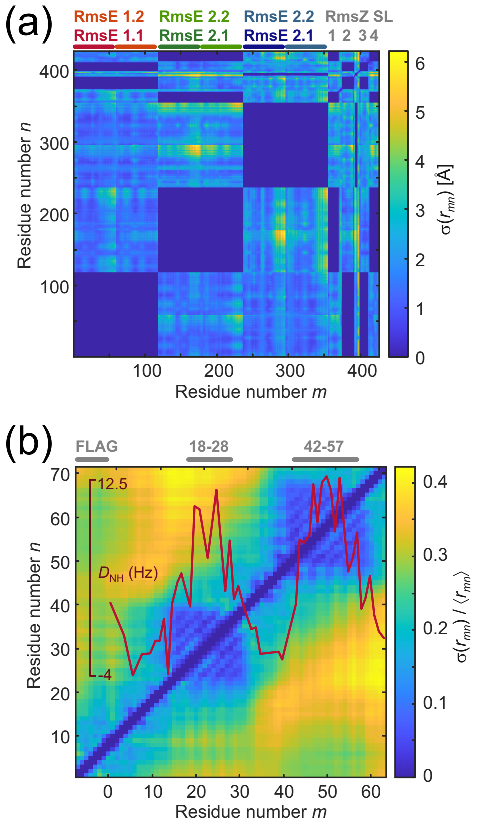

Fig. 2. Residue-pair correlation analysis for RsmE/RsmZ [5] (a) and the NMR/SAXS ensemble of PaaA2 [6] (b). The PaaA2 construct contains an N-terminal FLAG tag (residues $-7 \ldots 0$). The crimson curve and data points in (b) are N-HN NMR residual dipolar couplings taken from [6].

start MMM. In the File menu select the New from PDB/local item to load the PDB structure 2MF1_rigid_bodies_unseparated.pdb. In the Utilities menu select the Separate rigid bodies item and then the restraint file RsmE_RsmZ_RigiFlex_restraints.dat. This generates a rigid body file RSM0.pdb, as specified in the # PDB line of the restraint file.

In the Build menu select the RigiFlex item, click the Restraints button in the RigiFlex window and again select the restraint file RsmE_RsmZ_RigiFlex_restraints.dat. After the template file has been loaded and the spin label rotamers generated, click the Rigi button. Confirm the suggested name RSM1 for the output. This searches for valid RBAs and should be finished after a few minutes. Upon completion, the button changes to FlexRNA. Click it. Progress is displayed in the **Runtime information** panel. Completion can take an hour or so. The individual models and a report on RNA linker generation are automatically saved. Close the RigiFlex window.

In order to generate an ensemble and check the restraint fit, select the item EnsembleFit in the Ensembles menu. Click Load restraints to load the restraint file RsmE_RsmZ_RigiFlex_restraints.dat. Click Make conformer list. This will suggest the name RSM1_m*.pdb for the individual conformer files. Confirm this. The Make conformer list and Load conformer list pushbuttons will grey out. Click the Run iteration button. All restraints are evaluated by computing spin label rotamers and distance distributions, but the populations are not fitted, since # NOFIT is specified in this restraint file. Instead, uniform populations are assumed. Once the ensemble is generated a report window opens that shows the file list and the Save & test ensemble button becomes available. Click it. When this is completed, a plot of pair RMSD between conformers is displayed, and individual Matlab figures visualize how well the distance distribution restraints (DDRs) are fitted. You can ignore the error messages in the Message board panel of the main MMM window that stem from missing experimental DEER data. You can now close the EnsembleFit window.

Use the New from PDB/local item from the File menu to load the ensemble RSM1_conformers_ensemble_ordered.pdb and the Run script item from the Jobs menu to load the MMM script color_RsmE_RsmZ.mmm. This generates the view in the top panel of Figure 4(b) of the main text. In order to generate the view in the bottom panel, type camrotate 0 90 in the **Command line** of MMM.

B. *Generation of a representative ensemble for RsmE/RsmZ*

In the Matlab command window, change to the MMM subdirectory demo\RsmE_RsmZ and start MMM. If you did not do that before, generate the PDB file RSM0.pdb with separated rigid bodies as described in Section III-A. In the Build menu select the RigiFlex item, click the Restraints button in the RigiFlex window and select the restraint file RsmE_RsmZ_Rigi_restraints.dat. Click on the Rigi pushbutton. Rigi will not ask for an output file name, but will merely generate a solution list RsmE_RsmZ_400000_solutions.dat. This name is specified in the restraint file, with _solutions.dat being automatically appended. The RBA solution search will run for about half an hour. Close the RigiFlex window after the Search has finished. Close and restart MMM.

Again in the Build menu select the RigiFlex item, click the Restraints button in the RigiFlex window, but now select the restraint file RsmE_RsmZ_Flex_restraints.dat. Click the RigiFlex button that will process the solution list obtained before and will generate the flexible RNA linkers. RigiFlex asks for an output file name and suggests RMS1.pdb. Change this to RMS1_large.pdb. The solution list is processed within a few minutes, but generation of all RNA linkers can take more than a day of computation time in this case.

Once it is completed, close the RigiFlex window and MMM and restart MMM. Use the item EnsembleFit in the Ensembles menu. Click Load restraints to load the restraint file RsmE_RsmZ_Flex_restraints.dat and then Make conformer list. Change the suggested file name pattern to RSM1_large_m*.pdb. Activate the autoiterate checkbox and click Run iteration. Spin

label rotamers and distance distributions are now computed. Progress is displayed in the field `Evaluation` of the panel `Ensemble diagnostics`. After Evaluation, EnsembleFit starts population fitting. Progress can be followed in `Ensemble diagnostics` panel. After population fitting has finished for all blocks of conformers, `Iteration` is replaced by `Completed`. Click `Save & test ensemble`. After completion, close the `EnsembleFit` window.

Use the `New from PDB/local` item from the `File` menu to load the ensemble `RSM1_large_conformers_ensemble_ordered.pdb` and then twice the `Run script` item from the `Jobs` menu to load the MMM scripts `color_RsmE_RsmZ.mmm` and `RSM1_larger_conformers_ensemble_ordered_transparency.mmm`. This generates the view in the top panel of Figure 4(c) of the main text. For the view in the bottom panel, type `camrotate 0 90` in the **`Command line`** of MMM. The ensemble can slightly differ from the one shown in the main text, since RNA linker generation is not deterministic.

IV. Ensemble Analysis

A. Pair correlation matrix for RsmE/RsmZ

In the Matlab command window, change to the MMM subdirectory `demo\RsmE_RsmZ` and start MMM. In the `Ensembles` menu use the `EnsembleAnalysis` item. Load the ensemble `RSM1_ensemble_std_view.dat` with the `Load ensemble 1` button. After the ensemble is loaded, SortGroup is performed and the pairwise RMSD matrix is displayed. For the case at hand, you obtain the plot shown in Figure 7(a) of the main text. By clicking on the `Copy` button, you can detach it as a separate Matlab figure for printing, saving in a graphics format, or copying to the clipboard.

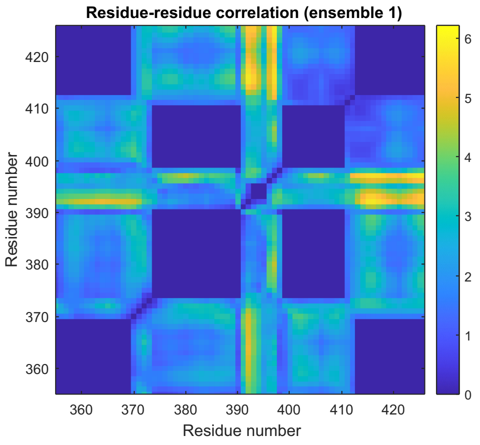

Fig. 3. Correlation between RNA segments in the RsmE/RsmZ ensemble generated with RigiFlex and EnsembleFit based on restraints that assume the structure reported by Duss *et al.* [3] as the ground truth.

Click the button `Compute correlation 1`. After completion, the plot will change to the one shown in Figure 7(b) of the main text. Again click the `Copy` button. Type `axis([355,426,355,426]);` in the Matlab command window. This will display only the RNA part, as shown in Figure 3. Stemloops 3 (residue numbers 399-410) and 4 (413-426) are somewhat more strongly correlated than other stemloop pairs, except of course for stemloops 1 and 4 which belong to the same rigid body. The binding motif in the linker between stemloops 2 and 3 (residue numbers 393-395) is not as much disordered with respect to stemloop 4 as the other sections of this linker.

B. Sorting and assembling the ensemble of PaaA2

In the Matlab command window, change to the MMM subdirectory `demo\PED5AAA` and start MMM. The pair correlation matrix for PaaA2 (PED5AAA) can be visualized in the same way as described for RsmE/RsmZ by loading the ensemble description `pEDB_5AAA_ensemble.dat` in EnsembleAnalysis. In this case, no complete PDB file is provided for the ensemble, but rather the individual PDB files listed in `pEDB_5AAA_ensemble.dat`. Uniform population of the 50 conformers is assumed. After EnsembleAnalysis has assembled and sorted the ensemble, the plot appears that is shown in Figure 7(e) of the main text. Click `Compute correlation 1`. The $\sigma(r_{mn})$ correlation plot that is initially shown is somewhat less clearly structured than the one shown in Figure 7(f) of the main text. To obtain the latter one, select `1/r` in the **`Normalization`** panel. Normalization to $1/\sqrt{r}$ is also offered and can be useful as it corresponds to random coil behavior in an ideal solvent. However, compaction analysis (vide infra) is better suited for detecting weak deviations from random-coil behavior and more general regarding scaling exponents.

The sorted ensemble can be saved as a single PDB file by clicking `Save 1`. The pair distance and pair correlation matrices as well as the ordering are automatically saved after computation as Matlab files *basis_name*`_pair_rmsd.mat` and *basis_name*`_correlations.mat`.

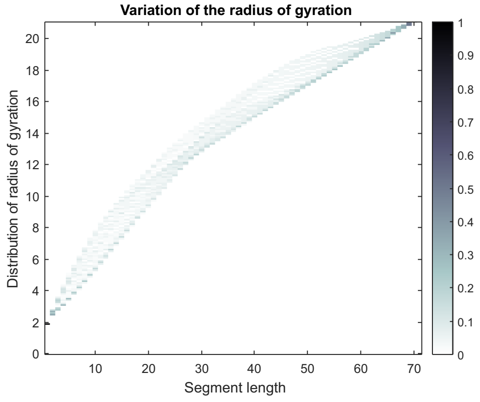

Fig. 4. In the ensemble structure of PaaA2, chain segments of different length differ in their distribution of the radius of gyration.

C. Compactness analysis of the ensemble of PaaA2

In the Matlab command window, change to the MMM subdirectory `demo\PED5AAA` and start MMM. In the `Ensembles` menu select the `LocalCompaction`

item. When prompted, load the ensemble description `pEDB_5AAA_ensemble.dat`. The LocalCompaction module generates five individual Matlab figures for each chain of an ensemble model. Four of these plots are shown in Figure 8 of the main text. The final plot, shown in Figure 4, visualizes the distribution of the radii of gyration as a function of segment length. It is similar to the plot shown in Figure 8(a) of the main text, with somewhat more detail.

V. RIGIFLEX AND ENSEMBLEFIT RESTRAINT FORMAT

A. General conventions for MMM restraint files

Keyword lines start with a hash `#` followed by one space and the keyword. Block keys are followed by lines that specify several restraints of the same type or provide additional parameters. A block key ends at the next keyword line or at the file end. It is good practice to conclude the restraint file with an `# END` key.

Block keys can have subkeys, which start with a colon character **:** followed without space by the keyword. A subkey can also be followed by several restraint lines. It ends when a new subkey or a new key or the file end is encountered.

In the following, we print keys in black with `typewriter font`, their arguments in italic and *mediumblue*, subkeys in `dimgrey`, their arguments in *slateblue*, and restraint lines within a block key in *darkmagenta*. Optional arguments are in black parentheses. The possibility to specify several arguments of the same type is indicated by dots ...

The percent character `%` starts a comment. The remainder of the line after this character is ignored. As the current version of MMM features several distinct restraint readers for different modules, it is good practice to start restraint files with a declaration of their type. Relevant types in the context of this work are `# RIGIFLEX` and `# FLEX`. The latter restraint file type is for the standalone version of Flex.[7] Declaration keys are not strictly required for running Flex or RigiFlex, but they are required when using the same restraint file for EnsembleFit.

B. Structure and model information

`# PDB` *rigid-body-file* (*output-identifier*)

A file *rigid-body-file*.pdb must exist and must specify atomic structure of all rigid bodies (vide infra). A four-character pseudo-PDB identifier *output-identifier* should be specified for output PDB files. It defaults to RIFL.

`# RIGID` *chain-ID-1* (*chain-ID-2* ...)

This block key can occur multiple times. Each instance defines one rigid body by specifying at least one identifier *chain-ID-1* of a chain in the rigid-body template PDB file that belongs to this rigid body. As many additional chain identifiers can follow as is necessary. Chain identifiers must be in parentheses, i.e. `(A)` for chain A.

The following lines in the block specify three reference sites in this rigid body:

adr (*label*)

Here, *adr* is a residue address, such as `(A)8` for residue 8 in chain A and *label* denotes a spin label, such as `MTSL` (methanethiosulfonate spin label). The label specification defaults to MTSL. Exactly three reference sites must be specified. Otherwise the restraint file is not further processed.

`# RNA` *chain-ID* (*maximum-time*)

This key defines an RNA chain. In the current implementation only one RNA chain can be generated. The chain identifier *chain-ID* is used only at an intermediate stage, but is a mandatory argument. It must be distinct from chain identifiers in the rigid-body template. The chain identifier must be in parentheses, i.e. `(R)` for chain R. Argument *maximum-time* is optional and defaults to one hour. It specifies a maximum computation time for each flexible RNA segment between binding sites.

Subkeys `:SEQ` and `:BIND` are mandatory, whereas subkey `:MODELS` is optional.

Subkey specifications are:

`:SEQ` *nt-1 nt-2 sequence*

Here, *nt-1* is the residue number for the first nucleotide, *nt-2* the one for the last nucleotide, and *sequence* the sequence in single-letter format.

`:BIND`

is a block subkey without any arguments. Each line in the block specifies one binding motif.

nt-1 adr-1 nt-2 adr-2

Here, *nt-1* is the residue number for the first nucleotide of a binding motif in the output PDB file, which must match the sequence specification in the `:SEQ` subkey. The MMM residue address *adr-1* specifies this nucleotide in the rigid-body template. In analogy, *nt-2* and *adr-2* pertain to the last nucleotide in the binding motif.

`:MODELS` *number* (*probability*)

Here, *number* is number of flexible linker conformers to be computed and *probability* the probability statistically covered by the conformers (see caption of main text Figure 2). The defaults are 1 and 0.5, respectively, if the subkey or the second argument is missing. We do not currently recommend to change them.

`# PFLEX` *res-1 res-2 N-anchor C-anchor*

This block key defines one flexible peptide segment. Multiple `# PFLEX` blocks are allowed. Arguments *res-1* and *res-2* are residue numbers of the last and first residue in the flexible segment, whereas arguments *N-anchor* and *C-anchor* specify the N-terminal and C-terminal anchor residues in a rigid body by MMM residue addresses.

Subkey specifications are:

`:SEQ` *sequence*

where *sequence* is the peptide sequence in single-letter format. This subkey is mandatory, the following ones are optional.

:MODELS *number* (*probability*)

Here, *number* is number of flexible linker conformers to be computed and *probability* the probability statistically covered by the conformers (see caption of main text Figure 2). The defaults are 1 and 0.5, respectively, if the subkey or the second argument is missing. We do not currently recommend to change them.

:TIME *max-time*

where *max-time* is the maximum computation time for one segment in hours (defaults to 1 h).

:DEER *label-1* (*label-2*)

where *label-1* specifies the spin label (mandatory, if a :DEER subkey is present). For orthogonal spin labelling, the second argument *label-2* specifies the second label.

:DEER is a block subkey with each line in the block specifying one DDR:

adr-1 adr-2 mean std (*exp-distr*)

where *adr-1* and *adr-2* are addresses of the labelling sites. For labelling sites inside the flexible segment, they are residue numbers that relate to arguments *res-1* and *res-2* of the # PFLEX key. Furthermore, *mean* and *std* are the mean distance and standard deviation of a Gaussian restraint. If both numbers are negative, they are interpreted as lower and upper bound. If they are zero, the distribution is computed, but the restraint is not included in modelling. The default unit is nanometers. This can be changed with the # UNITS key.

The optional argument *exp-distr* specifies the file name of a distance distribution file, which is expected in a subdirectory DeerLab of the current directory and must have the format of a distance distribution output file of DeerAnalysis. Full experimental distributions are currently only used by EnsembleFit, but not by RigiFlex or Flex.

Several :DEER blocks for different labels or label combinations are allowed.

The # PFLEX key allows for further subkeys that match all allowed restraint definitions of Flex [7] (replace # by :). In particular, the subkeys :OLIGOMER, :DEPTH, :HELICES, :STRANDS, :CISPEPTIDE, :APROP, :BPROP, and :CPROP are implemented.

C. Further restraint definitions

MAXSIZE *maximum-extension*

This key allows to set a maximum upper bound *maximum-extension* for unspecified restraints between reference points in rigid bodies. The default is 18 nm. The default unit is nanometers and can be changed by key # UNITS.

DEER *label*

This block key defines DEER restraints (DDRs), with the same spin label at both sites. Multiple # DEER blocks are allowed for using different labels. Argument *label* specifies the label. However, if both sites reference sites are defined in a # RIGID key, they must always use the same label. The following lines in the block define distance distribution restraints. They have the same format as specified above for the :DEER subkey of the # PFLEX key:

adr-1 adr-2 mean std (*exp-distr*)

ODEER *label-1 label-2*

This block key is used for DEER restraints (DDRs), with the different spin labels at the two sites. Multiple # ODEER blocks are allowed to allow for using different label combinations. Arguments *label-1* and *label-2* specify the labels. The following lines in this block key are analogous to the # DEER key.

SANS *data* (*resfile*) (*D_2O-content*)

This key specifies a small-angle neutron scattering restraint that is processed by CRYSON from the ATSAS package,[8] which must be installed and on the Matlab path. Argument *data* specifies the data file, optional argument *resfile* the resolution file, and optional argument *D_2O-content* a fraction of heavy water in the buffer. If *D_2O-content* is specified, a resolution file must also be specified (this is good practice anyway). Several # SANS restraints are allowed, for instance, with different detector distance or heavy-water content.

SAXS *data* (*sm*)

This key specifies a small-angle x-ray scattering restraint that is processed by CRYSOL from the ATSAS package,[8] which must be installed and on the Matlab path. Argument *data* specifies the data file, optional argument *sm* specifies a maximum scattering vector in inverse Å up to which the curve is fitted. By default, the whole curve ist fitted.

CROSSLINK *linker*

This block key sets a crosslink restraint with argument *linker* specifying a linker type. The type information is only used to distinguish several crosslink restraints for the same site pair. The following lines specify crosslinked residue pairs in a protein or complex:

adr-1 adr-2 max-distance

Here, *adr-1* and *adr-2* are MMM addresses of the crosslinked sites, which must be peptide residues (must have a Cα atom). Argument *max-distance* specifies the maximum allowed distance between the Cα atoms in Å. This value can be overwritten in the user interface, but then the same value is used for all crosslink restraints. In the user interface, it can also be specified which percentage of the crosslink restraints must be fulfilled for any acceptable RBA. Crosslink restraints are processed only by Rigi.

D. RigiFlex control

SAVERIGI

If this key is present, rigid-body arrangements (RBAs) are saved as PDB files during Rigi execution. By default, this is not done.

SEARCH

If this key is present, Rigi searches RBA space for valid solutions and stores a solution list without computing structures. This helps to avoid memory problems in cases where too many solutions would be encountered. It is also possible to process the whole solution list or part of the solution list in another MMM instance, i.e., to run search and processing partially in parallel on different computers. We strongly recommend to use the same Matlab version and CPU on both computers when doing this or when processing solution lists in general.

`# SOLUTIONS` *solution-file*

This key specifies the basis name for an RBA solution file by argument *solution-file*. The basis name is appended by `_solutions.dat`. In combination with the `# SEARCH` key, this is the output file name for solutions. If the `# SEARCH` key is missing, the solution file is read and only points in RBA space are considered, where solutions have been found in `# SEARCH` mode. This leads to a very fast Rigi run if the solutions are already known.

`# RIGIDISTR`

If this key is present, Rigi searches RBA space for valid solutions and stores a solution list without computing structures. This helps to avoid memory problems in cases where too many solutions would be encountered. It is also possible to process the whole solution list or part of the solution list in another MMM instance, i.e., to run search and processing partially in parallel on different computers. We strongly recommend to use the same Matlab version and CPU on both computers when doing this or when processing solution lists in general.

`# ENSEMBLE` *size probability*

The key specifies a maximum ensemble size *size* and a *probability* that is covered by the conformers in the ensemble (similar to the 50% probability covered by thermal ellipsoids in x-ray crystal structures). The probability is given as a fraction of 1. It can be overwritten in the graphical user interface and defaults to 0.6827, which corresponds on average to including a distribution width of twice the standard deviation.

`# MODELS` *max-number*

The key specifies a maximum number of RBAs to be generated by Rigi. This can be used instead of `# SEARCH` mode to avoid memory problems in initial runs and produce a given number of models for testing. In final production runs, the number should be sufficiently large for exhaustive search of RBA space. To limit the number of models in production runs, use `# MAXTRIALS` instead.

`# MAXTRIALS` *max-number*

The key specifies a maximum number of trials for the Rigi search of RBA space. Thus, it indirectly specifies sampling resolution of the exhaustive search. The largest number $\prod_{i=1}^{9n(n-1)/2} s_i$ (see main text) is used that is still smaller than *max-number*. The number of sampling intervals per core restraint (between reference points) is selected so that $\max(\Delta r_i)$ is minimized (best sampling resolution). The sampling resolution is displayed in the graphical user interface (RigiFlex window).

`# UNITS` [A|Angstroem|nm|nanometers]

This key allows to set units for DEER restraints and maximum size of the complex. The argument can take only the values indicated above. The default is nanometers.

E. *EnsembleFit control*

`# NOFIT`

If this key is present, the ensemble is only assembled, but not fitted to restraints. Uniform populations of all conformers are assumed. Use EnsembleAnalysys for generating restraint plots for such ensembles.

`# SUPERIMPOSE` *residue-range*

This allows for optimally superimposing all conformers in a certain residue range before saving the ensemble. The *residue-range* is specified by an MMM address, for instance `(A)182-283` for residues 182-283 in chain A. A warning is issued upon reading the restraint file if these residues do not exist in the rigid-body template for a RigiFlex run. No problem occurs if they do exist in the final models.

`# PLOTGROUP` *svg-color conformer-list*

This restraint is recognized only by EnsembleFit and leads to an (inconsequential) warning in RigiFlex. It allows to separate the distance distribution simulated for an ensemble into distributions for subensembles. The argument *svg-color* is an SVG color name, such as `darkblue`. The argument *conformer-list* is a comma-separated sequence of conformers to be included in this subensemble, such as `1,2,3,4,5,6,7,8`. Please note that the numbers refer to the sorted ensemble. The key is only used in a second run of EnsembleFit, after the ensemble or pair RMSD matrix have already been inspected. It is also processed when generating restraint fit plots with EnsembleAnalysis.

REFERENCES

[1] A. D. McLachlan, *J Mol Biol* **1979**, *128*, 49–79.
[2] C. T. Zahn, *IEEE Transactions on Computers C* **1971**, *20*, 68–86.
[3] O. Duss, E. Michel, M. Yulikov, M. Schubert, G. Jeschke, F. H. Allain, *Nature* **2014**, *509*, 588–92.
[4] A. D. Biasio, A. I. de Opakua, T. N. Cordeiro, M. Villate, N. Merino, N. Sibille, M. Lelli, T. Diercks, P. Bernado, F. J. Blanco, *Biophys J* **2014**, *106*, 865–874.
[5] O. Duss, M. Yulikov, F. H. Allain, G. Jeschke, *Methods Enzymol* **2015**, *558*, 279–331.
[6] Y. G. J. Sterckx, A. N. Volkov, W. F. Vranken, J. Kragelj, M. R. Jensen, L. Buts, A. Garcia-Pino, T. Jove, L. V. Melderen, M. Blackledge, N. A. J. van Nuland, R. Loris, *Structure* **2014**, *22*, 854–865.
[7] G. Jeschke, *Proteins* **2016**, *84*, 544–60.
[8] M. V. Petoukhov, D. Franke, A. V. Shkumatov, G. Tria, A. G. Kikhney, M. Gajda, C. Gorba, H. D. Mertens, P. V. Konarev, D. I. Svergun, *J Appl Crystallogr* **2012**, *45*, 342–350.
